# Bilateral-Asymmetry-Aware Multiple-Instance Learning for Breast Cancer Classification on Chest CT

**DOI:** 10.3390/life16081337

**Published:** 2026-08-14

**Authors:** Ioana-Andreea Cîrlig, Lucian-Mihai Florescu, Mădălin Mămuleanu, Cristina-Mihaela Ciofiac, Mihai-Alexandru Ene, Aurelia-Ștefania Domenco, Alexandru-Marian Olaru, Alexandra-Gabriela-Cosmina Țâru, Raluca-Elena Nica, Rossy-Vlăduț Teică, Ioana-Andreea Gheonea

**Affiliations:** 1 Doctoral School, University of Medicine and Pharmacy of Craiova, 200349 Craiova, Romania; andreea.cirlig97@gmail.com (I.-A.C.); cristina.ciofiac@umfcv.ro (C.-M.C.); mihai.ene@umfcv.ro (M.-A.E.); domenco.aura@gmail.com (A.-Ș.D.); 2 Department of Radiology and Medical Imaging, University of Medicine and Pharmacy of Craiova, 200349 Craiova, Romania; raluca.nica@umfcv.ro (R.-E.N.); rossy.teica@umfcv.ro (R.-V.T.); ioana.gheonea@umfcv.ro (I.-A.G.); 3 Department of Automatic Control and Electronics, University of Craiova, 200585 Craiova, Romania; 4 Department of Radiology and Medical Imaging, Emergency Clinical County Hospital of Craiova, 200642 Craiova, Romania; alex.olaru89@yahoo.com (A.-M.O.); alexandra.taru@yahoo.com (A.-G.-C.Ț.)

**Keywords:** breast cancer, chest CT, incidental findings, artificial intelligence, deep learning, multiple-instance learning, bilateral asymmetry, case-level classification, opportunistic screening

## Abstract

Incidental breast abnormalities may be visible on chest computed tomography (CT), although breast tissue is frequently outside the primary diagnostic focus of these examinations. This proof-of-concept study developed and internally evaluated a bilateral-asymmetry-aware multiple-instance learning framework (BAA-MIL) for case-level discrimination between breast cancer and non-malignant control examinations using preselected breast-containing axial chest CT images. The retrospective single-center case–control dataset included 89 CT examinations from 89 unique patients: 49 histopathologically confirmed breast cancer cases and 40 non-malignant control cases established according to predefined case-level reference criteria. The dataset comprised 304 selected axial CT images, yielding 608 unilateral breast-region crops arranged as 304 bilateral crop pairs. Paired breast crops were processed by a shared convolutional encoder, combined through an explicit side-symmetric asymmetry descriptor, and aggregated across axial images using gated attention-based multiple-instance learning. Performance was assessed using exploratory five-fold stratified patient-level cross-validation and a nested patient-level validation analysis. Under the primary nested patient-level validation protocol, the compact-encoder BAA-MIL implementation achieved an accuracy of 0.528 (95% CI, 0.427–0.629), balanced accuracy of 0.555 (95% CI, 0.465–0.637), sensitivity of 0.286 (95% CI, 0.160–0.415), specificity of 0.825 (95% CI, 0.694–0.930), and ROC AUC of 0.547 (95% CI, 0.420–0.665). The original non-nested analysis yielded higher exploratory estimates, but this analysis used the held-out folds for early stopping and checkpoint selection and is therefore reported only as secondary exploratory analysis. These findings support technical feasibility for a constrained case-level classification task using preselected chest CT images, but do not demonstrate detection of unsuspected incidental breast cancer across complete CT examinations. Performance estimates remain uncertain, and external validation using complete volumetric CT data is required before clinical use.

## 1. Introduction

Chest computed tomography (CT) is routinely performed for a broad range of clinical indications, with the entire breast region frequently included within the field of view of these examinations. Although CT is not a dedicated breast imaging modality, careful review of the breast region may identify incidental abnormalities requiring further evaluation, making recognition of these findings increasingly relevant for thoracic and general radiologists [1,2]. Suspicious CT-detected breast findings are important to report, as they may represent the first indication of an otherwise unsuspected breast cancer.

Several studies have emphasized the clinical relevance of incidental breast lesions detected on CT, particularly because lesions referred for dedicated breast assessment may prove malignant in a substantial proportion of cases. This is further supported by broader evidence on incidental imaging findings, with breast incidentalomas reported to have the highest malignancy proportion among organ-specific incidental findings in an umbrella review [3]. Moyle et al. found that 22 of 78 incidental breast lesions referred for breast assessment were malignant, with irregularity and spiculation being important predictors of malignancy [4]. CT examinations performed for non-breast indications may therefore provide the first opportunity to identify previously unknown primary breast cancers [5]. However, these findings may remain under-detected, as missed abnormalities in radiology can occur when lesions are unexpected or located outside the main diagnostic focus of the examination [6,7]. In chest CT interpretation, the breast represents a non-target region, which may make incidental breast lesions vulnerable to under-recognition.

The issue of under-recognition has also been demonstrated in studies specifically evaluating missed incidental breast cancers on chest CT. Park et al. found that 64.3% of incidental breast cancers visible on previous contrast-enhanced chest CT examinations had been missed in the original reports. Missed lesions were more frequently small or weakly enhancing, and treatment initiation was significantly delayed in the missed-lesion group [8]. In addition, Healey et al. reported a 17.3% cancer yield among abnormal breast findings initially identified on chest CT and followed by dedicated breast imaging, supporting the need for further evaluation of suspicious CT-detected breast abnormalities [9]. These findings suggest that improving the recognition and classification of breast abnormalities already visible on routine chest CT could have practical clinical value.

Artificial intelligence (AI) may offer a practical second-reader approach by automatically drawing attention to suspicious breast findings on CT images. Deep learning algorithms have already shown high sensitivity for breast cancer detection on chest CT in both internal and external test datasets [10]. More recent evidence suggests that AI-based workflows may identify more radiologically significant incidental breast lesions than original interpreting radiologists, supporting the potential role of AI as an assistive tool for opportunistic detection on examinations performed for other indications [11].

Despite these promising developments, CT-based breast lesion classification remains less explored than AI applications in dedicated breast imaging modalities, and routine chest CT examinations pose specific methodological challenges. Each examination contains multiple axial slices, only some of which may show diagnostically relevant breast findings, while the clinically meaningful decision is made at the case level rather than at the level of an isolated image crop. In addition, bilateral comparison is an important principle in breast imaging, as focal asymmetry between the two breasts may represent a suspicious finding. Therefore, an AI model designed for this setting should account both for the multi-slice structure of CT examinations and for inter-breast asymmetry, while avoiding image-level data leakage by keeping all slices from the same case within the same training or validation split.

In this proof-of-concept study, we aimed to develop and internally evaluate a bilateral-asymmetry-aware multiple-instance learning framework for case-level discrimination between histopathologically confirmed breast cancer and non-malignant control examinations using preselected breast-containing axial chest CT images. The proposed model integrates paired bilateral breast-region crops, an explicit inter-breast asymmetry descriptor, and attention-based aggregation of multiple axial images into a single case-level prediction. We explored whether the combination of bilateral information, explicit asymmetry encoding, and multiple-instance aggregation would provide numerically different discriminatory performance compared with simpler unilateral and non-asymmetry-aware baseline models. Model performance was evaluated using patient-level grouped internal cross-validation, with all images from the same patient maintained within the same data partition. This exploratory study assessed case-level classification within a radiologist-preselected image subset and did not evaluate detection of unsuspected incidental breast cancer or fully automated cancer detection across complete chest CT volumes.

## 2. Materials and Methods

### 2.1. Study Design and Setting

This retrospective, single-center, case–control, image-based proof-of-concept study was conducted using de-identified chest CT examinations obtained from the institutional imaging archive of the Medical Imaging Department of the University of Medicine and Pharmacy of Craiova. The study was designed to develop and internally evaluate a bilateral-asymmetry-aware multiple-instance learning framework for case-level discrimination between examinations from patients with breast cancer and non-malignant control examinations on chest CT. Because malignant and non-malignant cases were intentionally assembled to construct the study dataset, the investigation was considered a retrospective case–control study rather than a prevalence-based diagnostic cohort study.

### 2.2. Ethical Approval

The study was conducted in accordance with the principles of the Declaration of Helsinki and was approved by the Ethics Committee of the University of Medicine and Pharmacy of Craiova, Romania (Approval No. 124/02.06.2026). The requirement for individual informed consent was waived because of the retrospective design and the exclusive use of de-identified imaging and clinical data. All images were anonymized before dataset construction and model development, and no directly identifiable patient information was used during preprocessing, training, or evaluation.

### 2.3. Study Population and Eligibility Criteria

Potentially eligible examinations were identified from the institutional Picture Archiving and Communication System and available clinical/pathology records of the University of Medicine and Pharmacy of Craiova and were acquired between January 2023 and January 2025. The final study population comprised 89 chest CT examinations from 89 unique patients, including 49 cases of histopathologically confirmed breast cancer and 40 cases without evidence of breast malignancy according to the predefined reference standards described in Section 2.4. One CT examination was retained per patient. When more than one eligible CT examination was available, the earliest eligible examination was selected; for malignant cases, this was required to be a pretreatment staging CT.

After eligibility assessment, each included examination was assigned a unique anonymized study identifier to allow patient-level grouping throughout dataset construction and analysis.

Examinations were eligible if they: (1) included both breasts within the CT field of view; (2) had adequate image quality for preprocessing and model analysis; and (3) could be assigned a predefined case-level malignant or non-malignant reference label.

The malignant group included patients with histopathologically confirmed breast cancer and an available pretreatment chest CT examination performed for staging.

The non-malignant control group included patients without known breast malignancy or documented breast pathology at the time of CT, with no suspicious breast abnormality identified on the selected CT images and no subsequent diagnosis of breast cancer in the available institutional records during follow-up.

Examinations were excluded if they met any of the following criteria: (1) incomplete bilateral breast coverage; (2) severe motion or technical artifacts preventing reliable breast-region assessment or automated preprocessing; (3) unresolved or insufficient reference standard; (4) suspicious breast findings without histopathological clarification; (5) prior mastectomy, breast reconstruction, or post-treatment breast changes; or (6) initiation of breast cancer-directed treatment before the CT examination selected for analysis. The anatomical and treatment-related criteria were applied because these conditions could alter bilateral breast anatomy and potentially introduce confounding in an asymmetry-based model.

These exclusion criteria defined a restricted proof-of-concept population with adequate image quality, and a sufficiently established case-level reference standard.

### 2.4. Reference Standard and Outcome Definition

The reference standard was established at the patient/examination level. The target outcome was defined as a binary classification between breast cancer and non-malignant control status.

For malignant cases, the case-level reference label was based on histopathological confirmation of breast cancer. All patients had undergone mammography within 1 month before the pretreatment staging CT examination. The malignant label was assigned to the complete CT examination on the basis of the histopathological diagnosis. The CT images were not manually annotated to indicate the exact lesion location, involved slice, or affected breast side.

For non-malignant control examinations, the reference standard was based on the absence of known breast malignancy at the time of CT, the absence of suspicious breast findings on the selected CT images, and the absence of a subsequent breast cancer diagnosis in the available institutional records during a follow-up period of 6–12 months (median, 8 months). All control patients had undergone mammography within the 6 months preceding CT. Breast ultrasound was available only for a subset of control patients and was not performed systematically, while breast MRI was not routinely available in this group.

Cases were considered to have an unresolved or insufficient reference standard when the available clinical, imaging, histopathological, or follow-up information was not sufficient to assign a confident malignant or non-malignant case-level label.

The outcome label was assigned to the complete CT examination rather than to individual axial slices or breast crops. Consequently, during training, all selected axial slices and both unilateral breast-region crops from each malignant examination inherited the malignant case-level label, including crops from the contralateral breast that could be radiologically normal. These labels were used as weak case-level supervision and should not be interpreted as slice-level, breast-side-level, crop-level, or lesion-level malignancy annotations.

### 2.5. CT Image Source and Slice Selection

The chest CT examinations were acquired on a Siemens Biograph mCT 20-slice scanner (Siemens Healthineers, Erlangen, Germany) using the institutional arterial-phase contrast-enhanced chest CT protocol. Arterial-phase images were acquired approximately 30 s after intravenous contrast injection, according to the institutional protocol.

The imaging dataset consisted of de-identified two-dimensional axial chest CT images exported from the institutional imaging archive of the Medical Imaging Department of the University of Medicine and Pharmacy of Craiova as PNG files. The complete original DICOM series and associated acquisition metadata were unavailable for the present analysis.

The model was not trained or evaluated on complete chest CT volumes. Instead, each patient-level case consisted of a retrospectively selected subset of 2–7 axial PNG images containing bilateral breast tissue. These images were selected during dataset assembly by radiologists before preprocessing and model development. The present task should be interpreted as case-level classification using preselected breast-containing images, not as fully automated whole-volume breast cancer detection. In malignant cases, these preselected images were required to contain the known breast lesion; consequently, the task differs fundamentally from searching for an unsuspected incidental lesion in an unselected chest CT examination.

The axial images were selected retrospectively during dataset assembly by two radiologists before automated preprocessing and model development. For both malignant and non-malignant control cases, slice selection was based on anatomical inclusion of bilateral breast tissue within the selected axial images. This resulted in an asymmetric case-selection procedure: malignant cases were represented by breast-containing images that also included the known tumor, whereas non-malignant control cases were represented by breast-containing images without a target lesion. This design was appropriate for the constrained proof-of-concept classification task but may introduce spectrum and selection bias.

The source images used for model development were de-identified axial chest CT images exported from the institutional imaging archive in PNG format. The complete original DICOM volumes and acquisition metadata were not available for this analysis. PNG export was performed before model development, after de-identification, and no window-level adjustment, manual lesion annotation, or model-guided slice selection was applied during preprocessing. Because the analysis was based on exported PNG images rather than complete DICOM series, Hounsfield-unit calibration and full volumetric reconstruction were not available. This limitation is now explicitly acknowledged. The original DICOM volumes and export metadata were not available; therefore, the CT display window, bit depth, and exact intensity-scaling function used during PNG generation could not be reconstructed retrospectively. No Hounsfield-unit calibration was available after PNG export. The exported PNG images were reviewed during dataset assembly to ensure that the selected breast-containing images did not include visible burned-in patient identifiers. Orientation labels, acquisition overlays, or other burned-in display elements were not used as model inputs when present outside the automatically extracted breast-region crops. After export, all model inputs were generated from the cropped breast-region PNG images, resized to 224 × 224 pixels using the image-resizing procedure in the preprocessing pipeline, converted to three-channel RGB tensors, and normalized before model training.

No manual CT annotation was performed to indicate the exact lesion contour, lesion extent, or affected breast side as model input; therefore, the model was trained using only patient-level malignant or non-malignant labels.

No manual lesion segmentation, per-image crop correction, or model-guided image selection was performed during dataset construction. The selected axial images meeting the predefined bilateral breast-tissue inclusion criteria were retained. No slices were subsequently removed or added according to model predictions. The originating examination identifier and slice order were preserved to allow all images from the same patient to be grouped into a single multiple-instance case.

### 2.6. Dataset Composition and Unit of Analysis

Following application of the eligibility criteria and reference-standard requirements, the final dataset comprised 89 chest CT examinations from 89 unique patients. Of these, 49 examinations originated from patients with histopathologically confirmed breast cancer and 40 from non-malignant control patients without evidence of breast malignancy according to the predefined case-level reference standard described above.

The dataset included a total of 304 axial CT slices, comprising 156 slices from malignant cases and 148 slices from non-malignant control cases. Each axial image generated two unilateral breast-region crops, one from each breast side, resulting in 608 breast crops. Of these, 312 crops originated from malignant cases and 296 from non-malignant control cases. The number of axial slices available per examination ranged from 2 to 7, with a mean of 3.42 slices per case. From each axial image, two unilateral breast-region crops were generated, one for each breast side. Therefore, the dataset comprised 608 unilateral breast-region crops arranged as 304 paired bilateral crop sets.

The primary unit of analysis was the patient-level CT examination. Individual axial slices and breast crops were considered correlated components of their corresponding examination and were not treated as statistically independent observations. All axial images and bilateral crop pairs originating from the same patient were grouped throughout preprocessing, model training, internal validation, and statistical analysis.

The malignant or non-malignant outcome was assigned exclusively at the examination level. Therefore, crops originating from malignant examinations were not interpreted as individually malignant, because the contralateral breast could be normal and the target lesion was not treated as a manually annotated crop-level finding. The dataset consequently provided weak case-level supervision rather than slice-level, breast-side-level, crop-level, or lesion-level annotations.

### 2.7. Automated Breast-Region Extraction and Image Preprocessing

The analysis dataset was generated from the exported de-identified axial CT slices using a deterministic automated preprocessing script, without manual annotation or per-image editing.

For each axial slice, the script first loaded the image in grayscale and removed the surrounding air background using intensity thresholding. Morphological closing was then applied to fill internal low-density regions, including the lungs, and the largest external contour was extracted to approximate the body outline.

The anterior 35% of the body bounding box was isolated as the region most likely to contain breast tissue. This anterior region was divided vertically at the midline into two hemithoracic regions, corresponding to the two breast sides, denoted as Breast_A and Breast_B.

For each side, the tissue bounding box was trimmed by 20% per edge to reduce inclusion of adjacent structures. Each resulting breast-region crop was resized to 224 × 224 pixels. Image orientation was standardized before side assignment, allowing paired left–right breast crops from the same axial image to be reconstructed reliably.

After preprocessing, the generated crops were organized according to the case-level reference label and breast side, using the structure NON-MALIGNANT/MALIGNANT × Breast_A/Breast_B. The originating anonymized study identifier, examination identifier, slice identifier, and breast-side designation were preserved in the filenames, allowing reliable reconstruction of bilateral slice pairs and patient-level multiple-instance bags.

The preprocessing procedure was deterministic and fully automated. It did not use the malignant or non-malignant label to determine crop location, crop dimensions, or image transformations. Case-level labels were used only for dataset organization after crop generation and for subsequent model training. No manual breast segmentation, lesion segmentation, per-image crop adjustment, or replacement of automatically generated crop regions was performed. The same deterministic preprocessing pipeline was applied to all images before dataset partitioning and model training. Each axial PNG image was loaded in grayscale and thresholded using a fixed lower-intensity threshold of 15; pixels with intensity values greater than 15 were assigned to the foreground body mask. Morphological closing was then performed using an elliptical structuring element of 15 × 15 pixels for three iterations. External contours were extracted from the closed binary mask using cv2.RETR_EXTERNAL and cv2.CHAIN_APPROX_SIMPLE, and the largest contour by area was selected as the body contour. A filled body mask was then generated from this contour and used to define the body bounding box for subsequent anterior breast-region extraction.

Breast-region extraction was performed using a deterministic OpenCV-based preprocessing script. Each exported axial PNG image was loaded in grayscale. To remove the surrounding air background, pixels with intensity values greater than 15 were thresholded to create a binary body mask. Morphological closing was then applied using an elliptical structuring element of 15 × 15 pixels for three iterations to fill internal low-intensity regions, including lung spaces. External contours were extracted from the closed mask, and the largest external contour was selected as the body contour. A bounding box was computed around this contour. The anterior 35% of the body bounding box was retained as the candidate breast-containing region. This anterior region was split vertically at the midpoint of the body bounding box into two hemi-thoracic regions corresponding to the two breast sides. For each side, the non-zero mask pixels were used to compute a side-specific bounding box, which was then trimmed by 20% of its width and 20% of its height on each edge to reduce inclusion of skin, background, chest wall, and lung. Each resulting unilateral crop was resized to 224 × 224 pixels and saved as a PNG file. The script preserved the class label, anonymized case identifier, slice index, and breast-side designation in the output filename, allowing all crops from the same examination to be grouped during model training and cross-validation.

The anterior 35% crop and 20% per-edge trimming were predefined heuristic parameters chosen to retain the anterior chest wall/breast region while reducing inclusion of surrounding air, lung, and posterior thoracic structures. These values were fixed before model training and were applied identically to all images. They were not optimized separately for individual patients or according to model performance.

Preprocessing was implemented in Python 3.14.3 using OpenCV 4.13.0 and NumPy 2.4.4. Subsequent model development used PyTorch 2.11.0, torchvision 0.26.0, scikit-learn 1.8.0, Pillow 12.1.1, and Matplotlib 3.10.8.

While this automated preprocessing pipeline proved effective for the current analysis, it is not proposed as a universal gold standard. Its robustness and generalizability must be validated on larger, more diverse datasets before broader clinical or technical assumptions can be made.

### 2.8. BAA-MIL Model Architecture

The proposed model, termed BAA-MIL-bilateral-asymmetry-aware multiple-instance learning, was designed to integrate three clinically motivated elements: paired bilateral breast inputs, explicit inter-breast asymmetry encoding, and attention-based aggregation of multiple axial slices into a single case-level prediction. A complete layer-by-layer specification of the proposed BAA-MIL architecture, including encoder blocks, descriptor dimensions, dropout rates, gated-attention size, and classifier heads, is provided in Table 1.

Each convolutional block used a 3 × 3 convolution with stride 1 and padding 1, followed by BatchNorm2D, SiLU activation, and 2 × 2 max pooling with stride 2. Dropout2D was not applied after block 1 and was applied after blocks 2, 3, and 4 with rates of 0.1, 0.2, and 0.3, respectively. After adaptive average pooling and flattening, the encoder produced a 256-dimensional feature vector for each breast crop.

#### 2.8.1. Shared CNN Encoder

Each left and right breast crop was processed using a shared compact convolutional neural network encoder. The encoder consisted of four convolutional blocks with 3 × 3 convolutions and 32, 64, 128, and 256 channels, respectively. Batch normalization, SiLU activation, max-pooling, dropout, and global average pooling were used to produce a feature vector for each breast crop. Dropout was applied as Dropout2D with rates of 0.1, 0.2, and 0.3 after encoder blocks 2–4, respectively, and as standard dropout with a rate of 0.4 after the descriptor MLP activation.

For slice descriptor $h_{i}$, gated attention weights were computed as follows:

$$a_{i}=softmax\left(w^{T}\left[tanh\left(Vh_{i}\right)⊙sigmoid\left(Uh_{i}\right)\right]\right)$$

where V and U map the 256-dimensional slice descriptor to a 128-dimensional hidden attention space, and w maps this hidden representation to a scalar attention logit.

The same encoder weights were shared across breast sides and across slices, ensuring that left and right breast crops were processed in an identical feature space. For slice *i*, the left and right breast features are denoted as:

$$f_{i}^{L}=E\left(x_{i}^{L}\right),f_{i}^{R}=E\left(x_{i}^{R}\right)$$

where E represents the shared CNN encoder, and $x_{i}^{L}$ and $x_{i}^{R}$ represent the left and right breast crops of slice *i*.

An ImageNet-pretrained EfficientNet-B0 encoder was also evaluated as a backbone sensitivity analysis. Under the nested protocol, EfficientNet-B0 produced similar overall discrimination to the compact scratch encoder, with higher sensitivity but lower specificity. Therefore, the backbone comparison is interpreted as a sensitivity analysis rather than evidence of definitive encoder superiority.

For the EfficientNet-B0 comparison, the ImageNet-pretrained EfficientNet-B0 feature extractor from torchvision was used. The classification head was removed, and global adaptive average pooling produced a 1280-dimensional feature vector per crop. During training, the encoder was frozen for the initial 8 epochs. After this frozen phase, the last two top-level EfficientNet feature blocks were unfrozen and fine-tuned with a lower encoder learning rate of 1 × 10^−4^, while the descriptor and classifier heads were trained with a learning rate of 1 × 10^−3^. Batch-normalization layers in the pretrained encoder were kept in evaluation mode during fine-tuning to retain ImageNet running statistics. AdamW optimization with weight decay 1 × 10^−4^ and cosine-annealed learning-rate decay was used.

Therefore, the from-scratch CNN encoder was used in the final BAA-MIL model.

#### 2.8.2. Side-Symmetric Bilateral Asymmetry Descriptor

For each slice, the left and right breast feature vectors were combined into a side-symmetric bilateral descriptor. This descriptor incorporated both the bilateral mean appearance and the absolute inter-breast asymmetry magnitude:

$$s_{i}=\phi \left(\left[\frac{1}{2};\left(f_{i}^{L}+f_{i}^{R}\right)|f_{i}^{L}-f_{i}^{R}|\right]\right)$$

where ϕ represents a multilayer perceptron, $\frac{1}{2}\left(f_{i}^{L}+f_{i}^{R}\right)$ encodes the average bilateral appearance, and $|f_{i}^{L}-f_{i}^{R}|$ explicitly encodes inter-breast asymmetry.

This formulation is invariant to the arbitrary assignment of left and right breast labels, because both the mean feature and the absolute difference remain unchanged when the two sides are exchanged. The descriptor therefore encourages the model to learn relative inter-breast differences rather than absolute side-dependent features.

#### 2.8.3. Slice-Level Prediction and Gated Attention-MIL Aggregation

A shared linear slice-level head was used to generate a malignancy logit for each slice descriptor. The slice descriptors were then aggregated using gated attention multiple-instance learning. Attention weights were computed across the valid slices of each case and used to construct a case-level representation.

The model included both a slice-level prediction head and an attention-pooled case-level head. During training, the slice-level head provided the main supervision signal through case-label-propagated weak slice labels. The case-level head served as an auxiliary regularizer. During inference, the final case-level malignancy probability was obtained by attention-weighted aggregation of the slice-level malignancy probabilities:

$$p\left(\mathrm{MALIGNANT}|\mathrm{case}\right)=\sum_{i=1}^{S}a_{i}\sigma \left({\hat{y}}_{i}\right)$$

where $a_{i}$ is the attention weight assigned to slice *i*, ${\hat{y}}_{i}$ is the slice-level malignancy logit, σ is the logistic sigmoid function, and S is the number of slices in the case.

This inference rule was selected because it performed better than using the attention-pooled case head directly. Consequently, the case head was retained only as a training-time regularizer, whereas attention-weighted slice-probability aggregation was used for all reported predictions.

**Figure 1:**
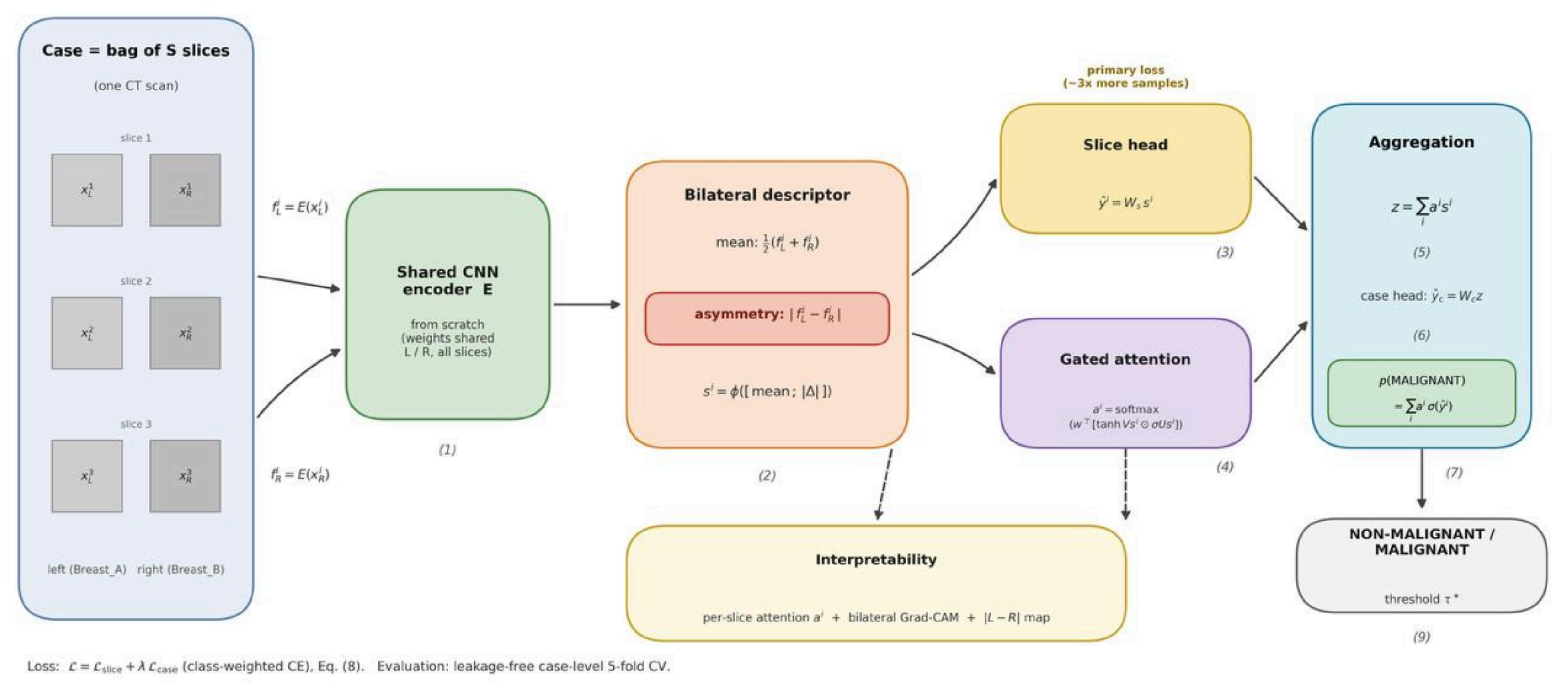
The BAA-MIL architecture. Each CT examination is represented as a bag of axial slices, with paired left and right breast crops extracted from each slice. A shared CNN encoder processes both breast sides, after which bilateral mean features and explicit inter-breast asymmetry features are combined into a side-symmetric descriptor. Slice-level predictions are aggregated using gated attention multiple-instance learning to generate the final case-level non-malignant/malignant prediction. Attention weights and bilateral Grad-CAM provide qualitative interpretability outputs.

The complete BAA-MIL workflow, including bilateral crop input, shared encoding, asymmetry-aware feature construction, slice-level prediction, gated attention aggregation, and case-level output, is summarized in Figure 1.

#### 2.8.4. Interpretability Outputs

Two qualitative interpretability outputs were generated for held-out cases. First, the gated attention weights were visualized across the slices of each case to indicate the relative contribution of each axial level to the final prediction. Second, bilateral Grad-CAM maps were generated for the top-attended slice, separately for the left and right breast crops. A left–right difference map was also produced to visualize the asymmetric regions contributing to the model prediction.

These interpretability outputs were used as qualitative explanations of model behavior. They were not treated as formal lesion-level annotations or as a substitute for expert radiological localization.

### 2.9. Training Strategy and Implementation Details

The model was trained separately within each fold of the cross-validation procedure using only the training cases from that fold. The final architecture used the compact from-scratch CNN encoder described above, followed by the side-symmetric bilateral descriptor, slice-level prediction head, gated attention-MIL module, and auxiliary case-level head.

The slice-level loss was based on weak case-label propagation, whereby each selected axial image pair inherited the non-malignant or malignant label of its parent examination. The case-level loss was used as a lighter-weighted auxiliary regularization term during training. The final training objective combined the slice-level and case-level losses.

The case-level auxiliary loss weight was set to λ = 0.5 to retain the slice-level weak-supervision loss as the primary training signal while still regularizing the case-level representation. This value was fixed across all folds and ablation variants.

The proposed method was implemented in PyTorch and trained using AdamW with an initial learning rate of 10^−3^, weight decay of 10^−4^, and cosine-annealed learning-rate decay over a maximum of 30 epochs. BAA-MIL was trained with mini-batches of 8 cases, with variable-length bags padded within each batch and masked during attention pooling; the per-crop CNN baseline used mini-batches of 16 crops. Early stopping was based on validation ROC AUC within each fold, with training stopped after 8 consecutive epochs without improvement and the best-AUC checkpoint restored for evaluation. The loss used inverse-frequency class-weighted cross-entropy, with class weights recomputed from the training cases in each fold as $N/\left(2n_{c}\right)$ for class c, combined as the slice-level weak-supervision loss plus a case-level auxiliary loss weighted by λ=0.5. All crops were resized to 224 × 224 pixels, converted to RGB, and normalized using ImageNet mean and standard deviation. During training, paired augmentation was applied identically to the left and right breast crops from the same slice: horizontal flipping with probability 0.5, rotation uniformly sampled from −12° to 12°, random zoom-crop with probability 0.5 and scale 0.85–1.00, and brightness/contrast factors uniformly sampled from 0.8 to 1.2. Vertical flips were excluded as anatomically implausible for axial CT images. The cross-validation split used random seed 42; for model initialization and stochastic augmentation, fold k used seed 42+k for NumPy and PyTorch. Final predictions were obtained by averaging predictions from the original and horizontally flipped inputs. Training was performed on a MacBook Pro with an Apple M3 Max chip and 36 GB memory using the Apple Metal backend.

### 2.10. Internal Cross-Validation and Patient-Level Partitioning

Model performance was evaluated using five-fold stratified cross-validation at the patient level, with stratification according to the malignant versus non-malignant outcome. All selected axial images and both breast-region crops originating from the same patient were assigned to the same cross-validation fold, thereby preventing direct patient-level overlap between the training and held-out partitions.

Within each fold, the held-out validation cases were used for early stopping and checkpoint selection. The operating threshold for threshold-dependent metrics was determined exclusively on the training partition predictions by maximizing balanced accuracy. The selected threshold was then applied without modification to the held-out fold. Each patient contributed one out-of-fold prediction for final performance aggregation. All performance analyses were conducted at the patient level, with all slices and bilateral crops from the same patient kept within the same partition. The original five-fold cross-validation analysis is reported as exploratory, whereas the nested patient-level validation analysis described below is reported as the primary bias-corrected evaluation.

Performance is summarized as the mean and standard deviation of the metrics obtained across the five held-out folds. As a complementary analysis, the held-out predictions were pooled across folds so that each patient contributed one out-of-fold prediction generated by a model that had not been trained on that patient. These pooled predictions were used to construct the confusion matrix and calculate case-level bootstrap confidence intervals.

#### Nested Validation, Early Stopping, and Threshold Selection

To address potential optimistic bias, model evaluation was repeated using a nested patient-level validation procedure. The outer loop used five-fold stratified cross-validation at the patient level. For each outer fold, the outer held-out cases were used only once for final performance evaluation. The remaining outer-training cases were further divided into an inner-training subset and an inner-validation subset using a stratified random split, with 20% of the outer-training cases assigned to inner validation. Models were trained only on the inner-training cases. Early stopping, checkpoint selection, and fold-specific operating-threshold selection were performed exclusively on the inner-validation cases. The selected checkpoint and threshold were then applied once to the untouched outer-test cases. All selected axial images and both breast crops from the same patient remained within the same split throughout this procedure.

### 2.11. Ablation Experiments

To assess the contribution of each architectural component, an incremental architectural ablation framework was evaluated under the same case-level cross-validation protocol. All models used the same patient-level fold structure and were compared at the case level.

The following model variants were tested:

**1. Baseline scratch:** a compact per-crop CNN applied independently to each crop, with crop predictions averaged to obtain a case-level prediction. This model did not use bilateral structure, explicit asymmetry encoding, or slice-level MIL aggregation.

**2. Single mean:** a single-breast slice-level model with mean pooling across slices, used to isolate the effect of using only one breast side.

**3. Bilateral mean:** a bilateral model using both breast sides fused by averaging, without an explicit asymmetry descriptor and with mean pooling across slices.

**4. Asymmetry mean:** a bilateral model incorporating the explicit $|f_{i}^{L}-f_{i}^{R}|$ asymmetry descriptor, with mean pooling across slices.

**5. BAA-MIL Full:** the proposed model, incorporating paired bilateral input, explicit asymmetry encoding, and gated attention-MIL slice aggregation.

This ablation design allowed the contribution of bilateral fusion, asymmetry encoding, and attention-based aggregation to be evaluated separately.

In the single-breast variant, the first crop stream, corresponding to Breast_A in the preprocessing output, was used for each slice; Breast_B was not provided to this model. Because the automatic cropper used side labels Breast_A and Breast_B rather than clinically verified anatomical laterality, this variant should be interpreted as a single-side input ablation rather than as a left- or right-breast-specific model. The exact input structure, feature construction, slice-aggregation method, and training settings for each ablation variant are summarized in Table 2.

### 2.12. Performance Metrics and Statistical Analysis

All performance metrics were computed at the case level. The primary performance metric was case-level ROC AUC. Secondary metrics included balanced accuracy, sensitivity, specificity, and accuracy.

Threshold-dependent metrics were calculated using the fold-specific operating thresholds. ROC AUC was treated as a threshold-free performance metric. Per-fold results are summarized as mean ± standard deviation across the five cross-validation folds.

For complementary pooled out-of-fold analysis, each case contributed one held-out prediction generated by the model from the fold in which that case was not used for training. Case-level bootstrap resampling with 2000 replicates was used to estimate 95% confidence intervals for accuracy, balanced accuracy, sensitivity, specificity, F1 score, and ROC AUC. Given the modest number of independent cases, confidence intervals were reported to emphasize the uncertainty surrounding the performance estimates.

Architecture, encoder-backbone, and inference-rule comparisons were not used to claim an unbiased estimate of a selected optimal pipeline. Because these comparisons were not embedded within a full inner-loop model-selection procedure, they are reported as exploratory or sensitivity analyses. The nested validation result is reported as the primary bias-corrected performance estimate for the primary compact-encoder BAA-MIL implementation.

## 3. Results

### 3.1. Dataset Composition and Cross-Validation Structure

The final analysis included 89 chest CT examinations from 89 unique patients, comprising 49 histopathologically confirmed breast cancer cases and 40 non-malignant control cases established according to the predefined reference standards. The main case-level cohort characteristics, including patient age, CT indication, breast density, malignant histology, tumor laterality, lesion-size range, selected axial images, and breast-region crops, are summarized in Table 3. The examinations contributed 304 selected axial CT images, including 156 from malignant cases and 148 from non-malignant control cases. Automated bilateral crop extraction generated 608 breast-region crops: 312 from malignant examinations and 296 from non-malignant control examinations. Each case contained 2–7 bilateral image pairs, with a mean of 3.42 pairs per case. No slice or crop was treated as an independent observation during model evaluation. Model performance was first evaluated using five-fold stratified cross-validation at the patient level as an exploratory original analysis. Because the held-out folds in this original analysis were also used for early stopping and checkpoint selection, these estimates were not strictly isolated from model optimization and are therefore reported as exploratory. To address this limitation, the primary performance analysis was repeated using the nested patient-level validation protocol described in Section Nested Validation, Early Stopping, and Threshold Selection, in which early stopping, checkpoint selection, and threshold selection were performed only within the outer-training partition before final evaluation on the untouched outer fold. Each patient contributed one out-of-fold prediction for final performance aggregation, and all slices and bilateral crops from the same patient remained within the same partition.

### 3.2. Ablation Analysis and Encoder Selection

The ablation analysis demonstrated numerical differences in case-level performance across the successive architectural variants, as summarized in Table 4. The baseline per-crop CNN achieved an accuracy of 0.616 and ROC AUC of 0.705. Using a single-breast slice-level model produced similar accuracy and a numerically higher ROC AUC of 0.743. Bilateral fusion was associated with a numerically higher accuracy of 0.640, while adding the explicit inter-breast asymmetry descriptor yielded an accuracy of 0.694 and specificity of 0.800. In this exploratory non-nested ablation analysis, the full BAA-MIL model, which combined paired bilateral input, explicit asymmetry encoding, and gated attention-MIL aggregation, showed the highest numerical performance among the evaluated variants; however, these ablation results are secondary to the primary nested validation analysis.

### 3.3. Uncertainty of Ablation Differences

Because the numerical differences between ablation variants were small relative to the cohort size, we additionally quantified paired patient-level uncertainty for each incremental model comparison, as summarized in Table 5. For each pair of models, performance differences were computed on the same outer-fold case predictions and 95% confidence intervals were estimated by paired bootstrap resampling at the patient level. These analyses showed that most performance differences had confidence intervals crossing zero, indicating that the ablation results should be interpreted as exploratory rather than as evidence of statistically definitive superiority of individual architectural components.

### 3.4. Primary Nested Validation Performance

Using the nested patient-level validation protocol, the primary BAA-MIL implementation with the compact scratch CNN encoder achieved a pooled out-of-fold accuracy of 0.528 (95% CI, 0.427–0.629), balanced accuracy of 0.555 (95% CI, 0.465–0.637), sensitivity of 0.286 (95% CI, 0.160–0.415), specificity of 0.825 (95% CI, 0.694–0.930), and ROC AUC of 0.547 (95% CI, 0.420–0.665). These estimates were lower than those obtained under the original non-nested cross-validation analysis, indicating that the original analysis likely provided an optimistic estimate of generalization performance (Table 6). The nested estimates are therefore reported as the primary bias-corrected performance results. As an exploratory high-specificity operating-point analysis, we also estimated sensitivity for the primary BAA-MIL model at fixed specificity levels of 0.90, 0.95, and 1.00. At specificity 0.90, sensitivity was 0.184 (95% CI, 0.023–0.386; threshold = 0.812). At specificity 0.95, sensitivity was 0.102 (95% CI, 0.000–0.292; threshold = 0.890). At specificity 1.00, sensitivity was 0.041 (95% CI, 0.000–0.180; threshold = 0.919). These results indicate that, in this small cohort, enforcing very high specificity substantially reduced sensitivity; therefore, these operating points should be interpreted as exploratory rather than clinically prescriptive.

### 3.5. Backbone Sensitivity Analysis

To evaluate whether the observed performance depended on the choice of feature extractor, we performed a backbone sensitivity analysis within the same BAA-MIL framework. The primary implementation used the compact CNN encoder trained from scratch, whereas the sensitivity analysis replaced this encoder with an ImageNet-pretrained EfficientNet-B0 feature extractor. All other components of the framework, including the bilateral descriptor, attention-MIL aggregation, nested patient-level cross-validation protocol, threshold selection procedure, and test-time augmentation, were kept unchanged. Therefore, this analysis isolates the effect of the encoder backbone rather than the bilateral multiple-instance framework itself. EfficientNet-B0 produced similar overall discrimination to the compact scratch encoder, with slightly higher accuracy, balanced accuracy, and ROC AUC, but with a different operating profile (Table 7). Sensitivity was higher with EfficientNet-B0, whereas specificity was higher with the compact scratch encoder. Paired bootstrap comparisons did not show a clear overall advantage for either backbone in accuracy, balanced accuracy, or ROC AUC; therefore, this comparison was interpreted as a sensitivity analysis rather than evidence of definitive encoder superiority.

### 3.6. Secondary Exploratory Non-Nested Performance

The original exploratory non-nested analysis BAA-MIL model achieved a mean case-level accuracy of 0.718, balanced accuracy of 0.726, sensitivity of 0.651, specificity of 0.800, and ROC AUC of 0.802 across the five held-out folds. Fold-level performance showed variability, with accuracy of 0.718 ± 0.121 and ROC AUC of 0.802 ± 0.099. These standard deviations describe variation across the cross-validation folds and should not be interpreted as independent-sample confidence intervals. In the complementary pooled out-of-fold analysis, accuracy was 0.719, with a case-level bootstrap 95% confidence interval of 0.618–0.809.

**Figure 2:**
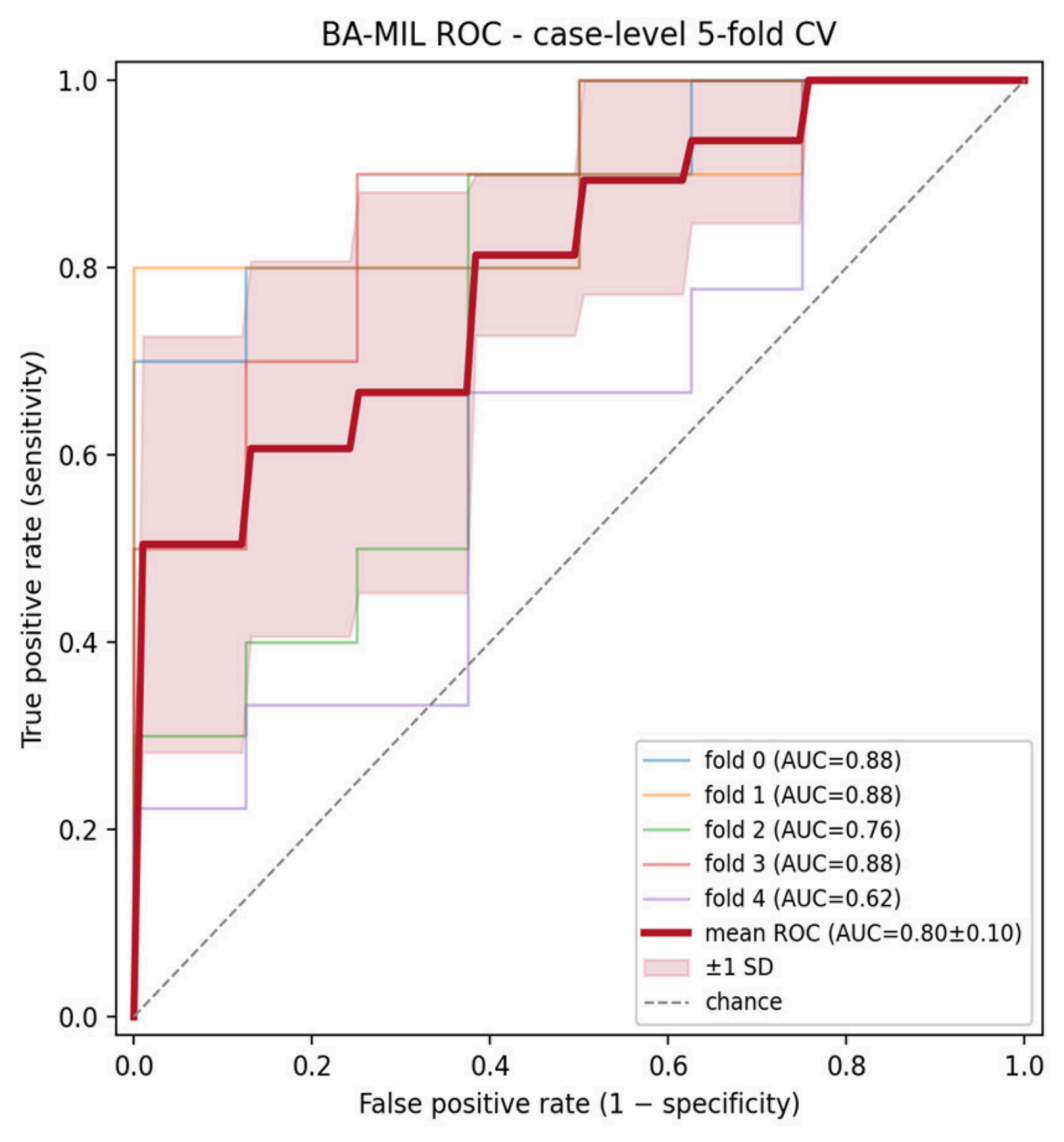
Receiver operating characteristic curve (exploratory original non-nested analysis). Per-fold ROC curves are shown with the mean ROC curve and ±1 standard deviation band across the five folds.

The ROC analysis is shown in Figure 2, with per-fold ROC curves and the mean ROC curve across the five cross-validation folds.

**Figure 3:**
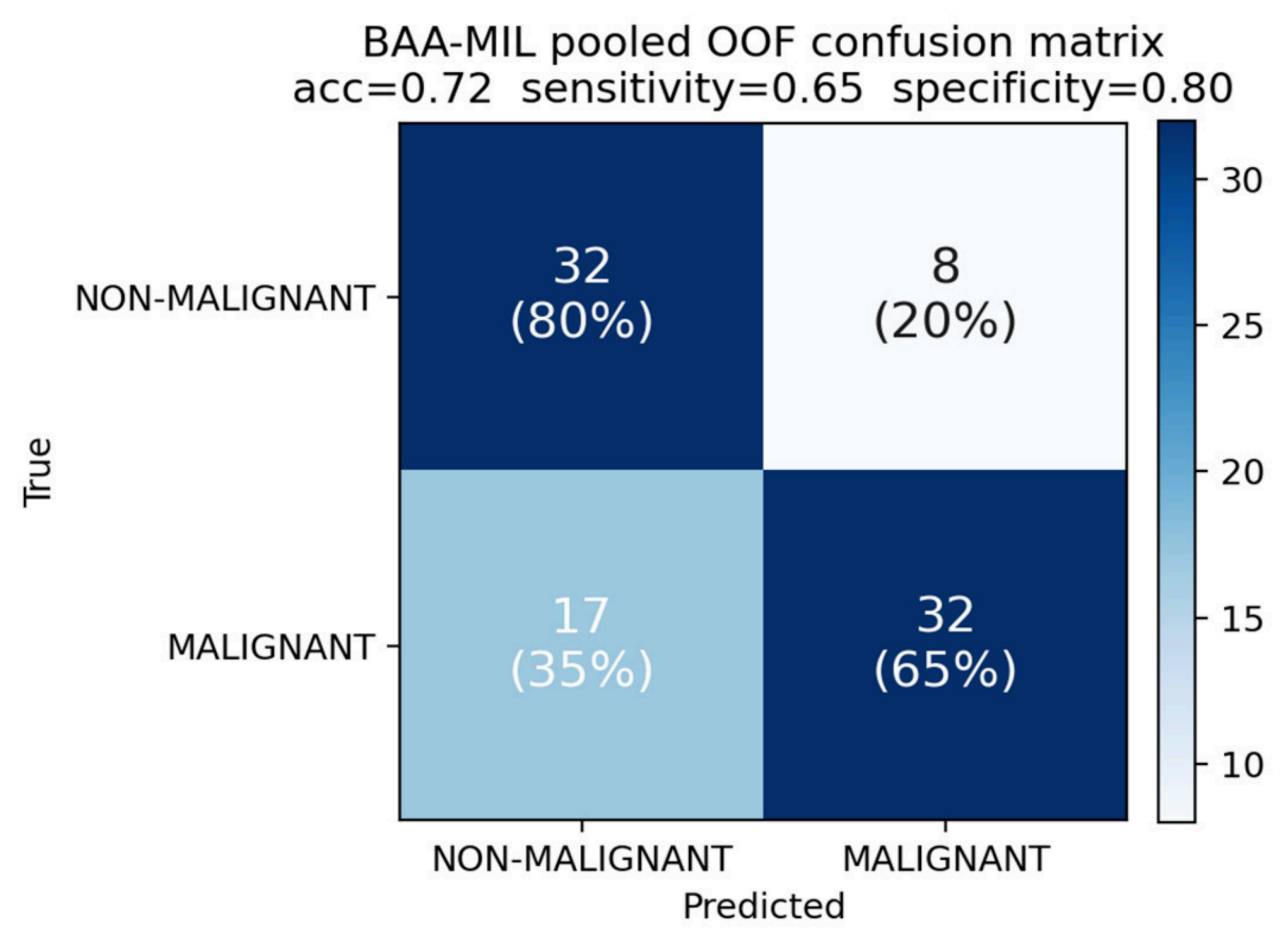
Pooled out-of-fold confusion matrix. Cases were binarized using the operating threshold selected within each corresponding fold. The matrix order is NON-MALIGNANT, MALIGNANT.

As a complementary pooled out-of-fold analysis, each case contributed one held-out prediction from the fold in which it was not used for training. Using fold-specific operating thresholds, the pooled out-of-fold accuracy was 0.719, with a 95% bootstrap confidence interval of 0.618–0.809. As shown in Figure 3, the pooled confusion matrix included 32/40 correctly classified non-malignant control cases and 32/49 correctly classified malignant cases, corresponding to 8 false-positive and 17 false-negative classifications.

### 3.7. Exploratory Inference-Rule Comparison

The trained BAA-MIL model allows two possible case-level read-outs: the attention-pooled case head and the attention-weighted slice-aggregation output. These two inference rules were compared using the same trained checkpoints and the same held-out folds, as summarized in Table 8.

Attention-weighted slice aggregation achieved numerically higher performance than the case head across all evaluated metrics. The case head achieved an accuracy of 0.684 ± 0.107, balanced accuracy of 0.683 ± 0.112, and ROC AUC of 0.764 ± 0.117. In comparison, slice aggregation showed numerically higher exploratory non-nested performance across these metrics. Attention-weighted slice aggregation achieved a numerically higher mean performance than the auxiliary case head across the held-out folds. Because both read-outs were compared using the same held-out predictions, this analysis should be considered an exploratory architectural comparison. The inference-rule comparison was performed after model training by comparing the auxiliary case-head output with attention-weighted aggregation of slice-level probabilities on the same held-out-fold predictions. Therefore, this comparison is post hoc and is reported only as an exploratory analysis. The attention-weighted slice-aggregation results should not be interpreted as an independently validated model-selection outcome.

### 3.8. Qualitative Interpretability Analysis

**Figure 4:**
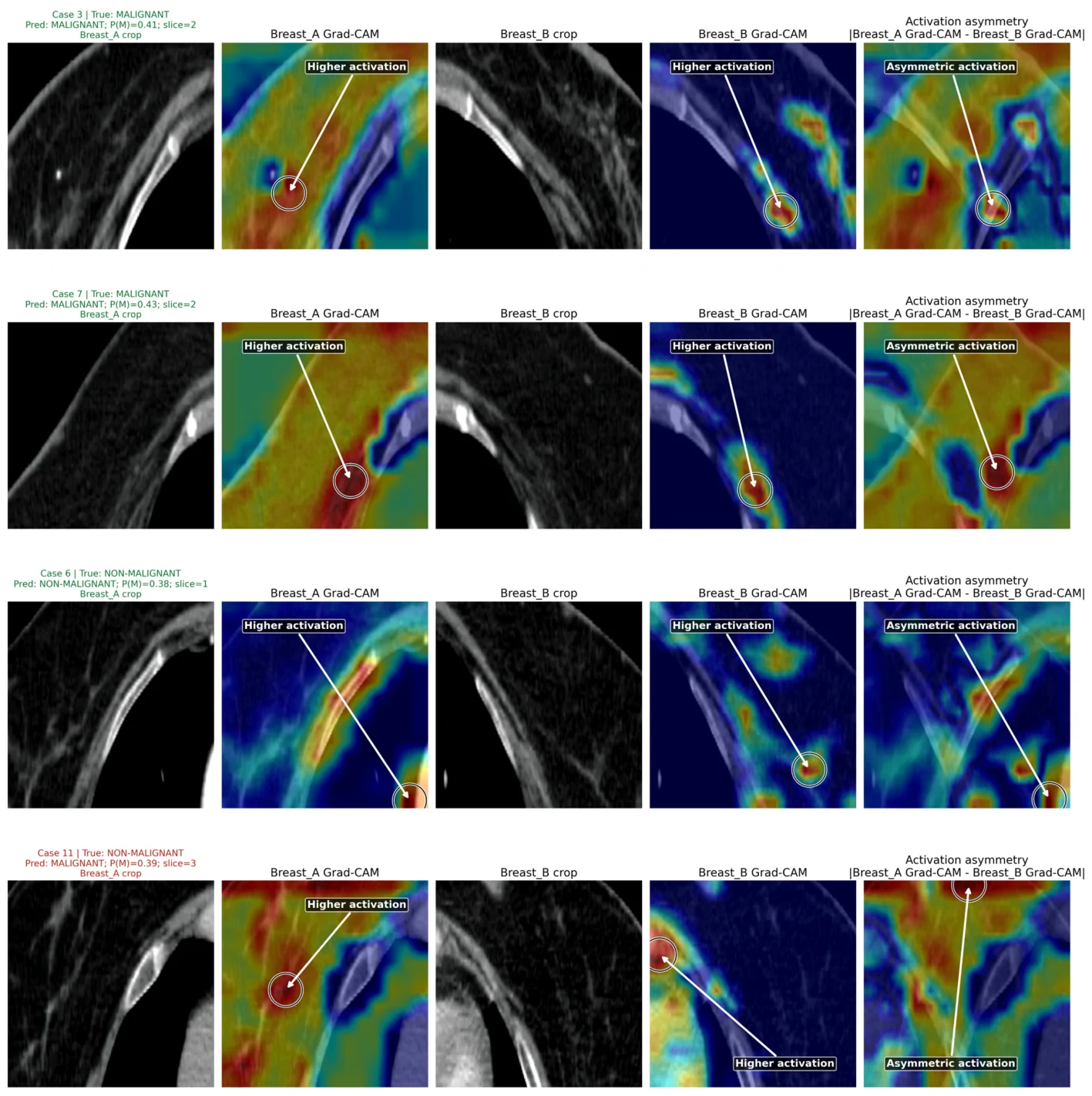
Preprocessing-aware interpretability examples from held-out cases.

Qualitative interpretability was assessed using bilateral Grad-CAM maps and per-slice attention weights. Bilateral Grad-CAM was generated for the top-attended slice of held-out cases. The activation maps localized to breast soft-tissue regions, while the left-right difference maps highlighted asymmetric regions contributing to the model prediction, supporting the rationale for incorporating explicit inter-breast asymmetry into the model architecture (Figure 4).

For each case, the original axial CT slice is shown alongside the automatically extracted paired breast-region crops used as model input. Grad-CAM was applied to these model inputs rather than to manually corrected regions, so the visualizations reflect the complete inference pipeline, including limitations of automatic crop extraction. These examples are presented only as qualitative audits of model behavior. Because no lesion contours, lesion-side labels, or slice-level lesion annotations were available, no quantitative localization metric, such as overlap with lesion segmentation or distance from lesion center, could be calculated. Therefore, Grad-CAM should not be interpreted as evidence of precise lesion localization. In several examples, activation is concentrated over asymmetric soft-tissue regions, which is consistent with the bilateral-asymmetry hypothesis underlying BAA-MIL. However, the visualizations also reveal that the automatic cropper may incompletely capture breast tissue or include adjacent thoracic structures. Per-slice attention weights were reviewed qualitatively but were not retained as a main figure because they were generally only mildly non-uniform. Attention was therefore interpreted as a case-level aggregation mechanism rather than as a slice-localization method. These attention distributions were only mildly non-uniform, which is expected given that most cases contained only three to four slices and that the bilateral descriptor already summarized each slice. Therefore, the attention mechanism should not be interpreted as precise slice-level lesion localization. Instead, its contribution appears to be mainly related to regularized aggregation of slice-level predictions within the case-level MIL framework.

## 4. Discussion

### 4.1. Principal Findings

This study developed and evaluated a bilateral-asymmetry-aware multiple-instance learning framework for case-level discrimination between breast cancer and non-malignant control examinations on chest CT. The proposed BAA-MIL model was designed around two clinically relevant characteristics of this task: the need for bilateral comparison and the multi-slice structure of CT examinations. The exploratory ablation analysis suggested possible numerical differences between the proposed bilateral/asymmetry-aware MIL framework and simpler baseline models, but these differences should not be interpreted as establishing a definitive benefit of the asymmetry component.

The primary nested validation analysis yielded an ROC AUC of 0.547, with an accuracy of 0.528, balanced accuracy of 0.555, sensitivity of 0.286, and specificity of 0.825. The original non-nested analysis produced higher exploratory estimates, but these results are interpreted as secondary because the held-out folds were also used during model optimization. The ablation analyses are hypothesis-generating only. Although some non-nested metrics were numerically higher after adding bilateral information, explicit asymmetry encoding, and attention-based aggregation, these comparisons do not establish an independent diagnostic benefit of the asymmetry descriptor. In particular, ROC AUC did not improve after adding the asymmetry descriptor alone, and most paired uncertainty intervals crossed zero.

The performance profile should be interpreted as proof-of-concept rather than clinical readiness. Specificity was relatively strong, whereas sensitivity remained moderate, with 17 malignant cases misclassified in the pooled out-of-fold analysis. Therefore, the present model should not be viewed as a screening-ready system or as evidence of diagnostically relevant clinical performance, but as an exploratory methodological proof-of-concept for constrained case-level classification on preselected chest CT images.

### 4.2. Relation to Incidental Breast Assessment on Chest CT

Deep CNN models have demonstrated promising performance in medical data classification from CT images, supporting their broader application across CT-based diagnostic tasks [12]. In breast imaging, machine-learning and deep-learning methods have also been extensively investigated for lesion detection, classification, and diagnostic decision support across mammography, ultrasound, magnetic resonance imaging, histopathology, and thermography [13]. Data preprocessing and augmentation—including rotation, scaling, flipping, and intensity transformations—are commonly employed to increase training diversity and limit overfitting when annotated imaging datasets are small.

Transfer learning represents a frequently adopted strategy in breast imaging research, with models pretrained on large natural-image datasets subsequently adapted to modality-specific classification tasks [13]. Nevertheless, differences between natural images and grayscale CT data may limit the transferability of generic pretrained representations. This consideration is relevant to the present study, in which the compact CNN trained from scratch achieved numerically higher performance than the ImageNet-pretrained EfficientNet-B0 encoder. Sequential architectures have also been investigated for multi-slice CT analysis. In a thoracic CT application unrelated to breast imaging, Kara et al. combined CNN-based spatial feature extraction with a bidirectional long short-term memory network to model relationships between ordered CT slices [14]. Although developed for COVID-19 classification, this approach illustrates the broader potential value of integrating information across multiple axial images.

The present study is most directly related to work on incidental breast findings visible on chest CT rather than to the broader breast-imaging AI literature. Prior clinical studies have shown that breast lesions detected incidentally on chest CT can have meaningful malignant yield and may be missed when the breast is outside the primary diagnostic focus of the examination. Recent AI studies have begun to investigate whether automated systems can assist in identifying radiologically significant incidental breast lesions on chest CT. In this context, the current study should be interpreted as a proof-of-concept case-level classification framework using preselected breast-containing axial images, rather than as a fully automated whole-volume detection system. Direct comparison with previous studies remains difficult because published work differs in target task, imaging input, annotation strategy, and validation design. Some approaches evaluate lesion detection or radiologist-facing triage, whereas the present model evaluates patient-level discrimination between malignant and non-malignant examinations using weak case-level labels. This distinction is important because the model was not trained with lesion contours, breast-side labels, or complete volumetric DICOM examinations.

Recent breast-cancer AI studies have also addressed prognostic tasks outside CT imaging, including relapse prediction from histopathological images using ensemble machine-learning models [15]. Although such work illustrates the broader expansion of AI methods in breast oncology, it differs from the present study in imaging modality, clinical endpoint, annotation structure, and intended use.

Previous research has demonstrated the growing potential of artificial intelligence for identifying and characterizing malignancies on medical images. CNN-based systems have been applied to cancer detection and classification because of their ability to extract complex imaging features without requiring manually defined descriptors. Available reviews have reported encouraging performance across breast imaging modalities and have also highlighted the potential contribution of AI to the earlier identification of breast and other common cancers [16,17].

Nevertheless, direct comparison between the present findings and previously reported results is difficult. Published studies differ substantially in their target task, imaging modality, input definition, annotation strategy, dataset composition, and validation protocol. Some models perform lesion detection or segmentation, whereas the present framework addresses case-level discrimination between breast cancer and non-malignant control examinations. Furthermore, our model was evaluated using preselected breast-containing axial chest CT images rather than complete volumetric examinations or manually segmented lesions. Performance estimates from studies using lesion-centered inputs, complete volumes, image-level random splitting, or larger datasets are therefore not directly comparable with the primary nested ROC AUC of 0.547 obtained in the present study.

The present study also differs from conventional single-image classification approaches by treating each CT examination as a collection of correlated axial images and by explicitly incorporating bilateral comparison. Its main contribution is therefore not only the achieved discrimination but the development of an architecture aligned with the structure of the available data and with a clinically recognizable imaging cue. Accordingly, the results should be interpreted as exploratory proof-of-concept findings regarding bilateral-asymmetry-aware case-level modeling, not as evidence of diagnostically relevant performance or superiority over previously published breast AI systems.

Because prior studies differ substantially in clinical goal, imaging modality, annotation strategy, and validation design, direct numerical comparison is limited. To clarify the position of the present work within the literature, Table 9 summarizes representative related studies according to dataset size, modality, clinical objective, annotation type, validation strategy, reported performance, and relevance to the current approach.

### 4.3. Bilateral Asymmetry as a Clinically Grounded Model Component

A central feature of the proposed architecture is the explicit incorporation of bilateral asymmetry. Comparison between the two breasts is an established component of breast image interpretation because unilateral differences in tissue distribution, contour, or focal appearance may provide diagnostically relevant information. Instead of requiring the network to discover this relationship implicitly, BAA-MIL combines bilateral mean appearance with the absolute difference between left- and right-breast feature representations. The absolute-difference term is invariant to the arbitrary ordering of the two breast sides, encouraging the model to emphasize relative inter-breast differences rather than side-specific positional features.

Most previously described weakly supervised frameworks rely primarily on implicit feature learning, expecting the network to identify diagnostically relevant instance relationships from case-level labels. For example, constrained weak-supervision methods have been used to identify cancer-related regions in histopathology without comprehensive pixel-level annotations [18]. Multi-view attention approaches likewise aggregate complementary information from different views but do not necessarily encode an explicit, clinically defined difference between paired anatomical structures [19]. In contrast, the bilateral descriptor used in BAA-MIL introduces a task-specific inductive bias derived from the clinical practice of side-to-side breast comparison.

Other developments in MIL demonstrate alternative strategies for incorporating relationships between instances. AttriMIL uses multi-branch attribute scoring and spatial and slide-level constraints to improve attention discrimination in whole-slide histopathological image classification [20]. Geometric and graph-based approaches model spatial or topological relationships between image regions, thereby incorporating structural context into bag-level prediction [21]. These approaches are methodologically related to the present study in their use of structured inter-instance information, although they were not developed for bilateral breast comparison on chest CT.

The ablation results provide only exploratory and qualified support for the proposed asymmetry component. Adding the explicit asymmetry descriptor to the bilateral mean model increased accuracy from 0.640 to 0.694, balanced accuracy from 0.643 to 0.701, and specificity from 0.675 to 0.800. However, sensitivity decreased slightly from 0.611 to 0.602, and ROC AUC remained similar, changing from 0.743 to 0.741. The asymmetry descriptor therefore did not independently improve every performance metric. Its possible contribution was most evident in specificity and balanced accuracy, while the overall ablation pattern remained exploratory and hypothesis-generating.

### 4.4. Weak Supervision, Multiple-Instance Aggregation, and Encoder Selection

Attention-based MIL is appropriate when each case contains multiple correlated instances but only a case-level label is available. In this study, each CT examination was represented as a bag of selected axial image pairs, and attention was used to aggregate slice-level descriptors into a case-level prediction. However, the weak-supervision setup also introduced substantial label noise because all selected slices and both breast crops inherited the case-level label. In malignant examinations, this included contralateral breast crops that could be radiologically normal and slices that might not contain a visible tumor. As a result, the model may have learned case-level correlates or confounding features associated with the malignant cohort, rather than relying exclusively on lesion-specific imaging findings. The model should therefore be interpreted as a case-level classifier, not as a lesion-localization system. In addition, because malignant and control examinations may differ in clinical context, acquisition indication, contrast timing, patient habitus, or other case-level factors, weak case-level labels could permit the model to learn non-lesion-related correlates of group membership.

Comparable noisy-label and weak-supervision strategies have been applied in other medical imaging frameworks to reduce dependence on costly pixel- or instance-level annotations [22,23]. The number of independent observations in the present study nevertheless remained at 89 patients, regardless of the larger number of slice-level training instances.

Attention-based MIL provides a natural mechanism for aggregating variable numbers of potentially informative instances into a single case-level prediction. Gated attention mechanisms assign a relative contribution to each valid instance, allowing the model to construct a case representation without requiring every slice to be equally informative [19,24]. Related approaches include multi-view attention, attribute-aware MIL, graph-convolutional MIL, and attention-refinement strategies based on data augmentation [19,20,24,25]. The present model differs by combining attention-based case aggregation with an explicit bilateral descriptor tailored to breast anatomy.

The exploratory comparison between the two available inference rules showed numerically higher mean performance for attention-weighted aggregation of slice-level probabilities than for the auxiliary case head. Attention-weighted aggregation of slice-level probabilities achieved numerically higher accuracy, balanced accuracy, and ROC AUC than the attention-pooled auxiliary case head. The slice-aggregation output was consequently used for the final case predictions, while the case head functioned as an auxiliary training regularizer. Because this comparison was performed using the same trained checkpoints and held-out folds, it should be interpreted as an exploratory architectural analysis rather than definitive evidence that slice-probability aggregation is universally preferable.

The compact CNN trained from scratch also achieved numerically higher performance than the ImageNet-pretrained EfficientNet-B0 encoder. This finding may reflect the domain difference between natural color images and grayscale CT breast-region crops, as well as the potential advantage of a lower-capacity architecture in a small dataset. Previous research has similarly identified challenges in transferring representations between natural-image and medical-imaging domains [22]. Nevertheless, the present comparison was limited to one pretrained architecture, and broader conclusions regarding transfer learning would require evaluation of additional encoders and medical-image pretraining strategies.

### 4.5. Interpretability and Potential Clinical Relevance

The proposed framework provides two complementary qualitative interpretability outputs: bilateral Grad-CAM maps and per-slice attention weights. Grad-CAM and other explainable-AI methods, including SHAP and LIME, are increasingly used to visualize image regions that influence model predictions and to improve the transparency of medical imaging algorithms [26]. In the present study, Grad-CAM was applied directly to the automatically generated breast crops used by the network, rather than to manually corrected or retrospectively segmented regions. The resulting visualizations therefore provide an audit of the complete preprocessing and inference pipeline.

The activation maps were frequently concentrated within breast soft tissue, and the bilateral difference maps highlighted visually asymmetric regions. These observations are consistent with the architectural rationale underlying BAA-MIL. However, they do not establish that the network localized the histopathologically confirmed lesion or relied exclusively on pathology-specific imaging features. No lesion-level segmentation reference was available for quantitative localization assessment, and some crops incompletely captured breast tissue or included adjacent thoracic structures. Accordingly, Grad-CAM should be regarded as a qualitative model-audit tool rather than a lesion-localization method.

Per-slice attention weights were also only mildly non-uniform in several representative cases. Given that most examinations contained approximately three to four selected image pairs, attention may have functioned primarily as a learned aggregation mechanism rather than as a sharply selective lesion-localization process. High attention should therefore not be interpreted as evidence that a particular slice contains malignancy. Similarly, low attention does not establish the absence of diagnostically relevant tissue.

At the operating point used for pooled out-of-fold analysis, the model achieved a specificity of 0.800 but a sensitivity of 0.651, with 17 of 49 malignant cases classified as non-malignant. This error profile precludes autonomous diagnostic use and would be insufficient for a screening system intended to exclude malignancy. A potential future role, after validation on complete CT volumes, could involve opportunistic second-reader support for breast abnormalities visible on routine chest CT examinations. A lower decision threshold could theoretically prioritize sensitivity at the cost of additional false-positive findings, but any clinically meaningful operating point would require calibration and validation in a larger, consecutive external cohort. The current threshold should therefore be considered an exploratory operating choice rather than a recommended clinical threshold.

### 4.6. Methodological Strengths and Limitations

Several methodological strengths should be noted. First, the model architecture was designed around two characteristics relevant to chest CT breast assessment: bilateral comparison and the availability of multiple axial images per examination. Second, the patient-level examination was maintained as the primary unit of analysis. All slices and both breast crops from the same patient remained within the same cross-validation partition, preventing direct slice-level or crop-level overlap between training and held-out cases. Third, the incremental architectural ablation framework allowed the numerical contribution of bilateral input, explicit asymmetry, and attention-based aggregation to be examined separately. Finally, the preprocessing pipeline was automated and was evaluated in the form in which it was presented to the model, without manual correction of individual crops. This increased methodological transparency and allowed preprocessing failures to remain visible in the interpretability analysis.

The study also has important limitations. First, the cohort included only 89 independent patients, resulting in wide confidence intervals and noticeable fold-to-fold variability. The reported point estimates should therefore be interpreted together with their uncertainty and should not be considered precise estimates of clinical performance. Second, this was a retrospective, single-center, enriched case–control study without an external test cohort. Generalizability across patient populations, scanner manufacturers, reconstruction algorithms, acquisition protocols, institutions, and imaging settings remains unknown because no independent external or multicenter validation cohort was available. Because this was a case–control study with intentionally selected malignant and non-malignant control cases, the proportion of breast cancer cases was much higher than would be expected in routine chest CT practice. Therefore, the reported performance should not be interpreted as the expected performance in an unselected clinical CT population. Detailed subgroup-level indications for control CT examinations were not available for analysis; therefore, residual confounding by CT indication cannot be excluded.

The reference standard for the non-malignant control group was also subject to limitations. Although all control patients had undergone mammography within the 6 months preceding CT and had 6–12 months of available institutional follow-up, breast ultrasound was available only for a subset of patients and breast MRI was not routinely performed. In addition, diagnoses made outside the institution could not be comprehensively captured. Therefore, although the available imaging and follow-up strengthened the control-group designation, occult breast malignancy or a subsequent diagnosis outside the institution cannot be completely excluded.

Third, the analysis did not use complete volumetric DICOM examinations. Instead, each case contained a preselected subset of breast-containing axial images. This selection process creates a more constrained classification task than fully automated breast cancer detection across complete chest CT volumes and may introduce selection or spectrum bias. This bias was further increased by the asymmetric slice-selection procedure: malignant cases were required to include the known lesion, whereas control cases were selected only for anatomical inclusion of bilateral breast tissue. Consequently, the model may have learned differences related to the selected case spectrum rather than features that would generalize to unselected routine chest CT examinations. In particular, the model did not need to identify breast-containing slices within the full CT volume, reject irrelevant thoracic slices, or operate on complete DICOM examinations. Therefore, the present results should not be interpreted as evidence that the model can detect unsuspected incidental breast cancer in routine chest CT examinations.

Fourth, the model was trained using case-level weak supervision without independent breast-side, crop-level, or lesion-level labels. Consequently, malignant labels were propagated to all selected bilateral image pairs from malignant examinations, including the contralateral breast crop, which could be normal. Although MIL was used to tolerate this label uncertainty, the absence of detailed annotations for lesion contour, lesion extent, and affected breast side prevented quantitative lesion-localization analysis. The study also did not include a formal comparison with radiologists, and model performance should not be interpreted relative to human diagnostic performance. This weak-label design also creates a risk of case-level or acquisition-related confounding, because the model may exploit imaging or cohort differences associated with malignant versus control status rather than breast-lesion features alone.

Fifth, breast-region extraction was based on deterministic geometric assumptions rather than dedicated anatomical segmentation. Although quality control excluded non-evaluable bilateral pairs, without proper calibration and parameters, some retained crops could still omit peripheral breast tissue or contain chest wall, lung, or background structures. These imperfections may affect both classification and the appearance of Grad-CAM maps. Encoder selection, inference-rule selection, threshold determination, and early stopping must be interpreted within the internal cross-validation framework. Independent external evaluation is required to estimate performance in new clinical populations and acquisition settings. Because preprocessing was performed on exported PNG images rather than original DICOM data, the model did not use calibrated Hounsfield-unit values, acquisition metadata, or complete volumetric information. This may limit reproducibility across institutions and should be addressed in future work using complete DICOM examinations. In addition, due to the fact that body variables such as weight, height, and BMI were unavailable/incompletely available, we could not exclude confounding related to differences in patient habitus between malignant and control groups.

The exclusion criteria also limit the potential target population of the model. Because patients with prior mastectomy, breast reconstruction, post-treatment changes, severe artifacts, or unresolved breast findings were excluded, the results apply only to a selected subgroup of chest CT examinations with preserved bilateral breast anatomy and technically adequate images. In routine clinical practice, a fully automated system would need to handle these excluded scenarios or first identify examinations unsuitable for analysis.

### 4.7. Future Directions

Future work should prioritize evaluation in larger, consecutive, multicenter cohorts using complete DICOM chest CT examinations. A clinically deployable system would need automatic identification of breast-containing slices across the full volume, robust breast-region localization across scanners and acquisition protocols, and calibration in populations reflecting the low prevalence of incidental breast cancer on routine chest CT. Lesion-side, slice-level, and contour-level annotations would also allow quantitative assessment of localization and attention/Grad-CAM validity. Prospective studies should compare AI-assisted interpretation with radiologist interpretation and evaluate sensitivity, false-positive burden, probability calibration, threshold stability, recall recommendations, and downstream clinical impact.

This label-propagation strategy may encourage learning of patient-level or acquisition-related confounders, particularly in a small single-center case–control cohort, and future work should incorporate lesion-side, slice-level, and lesion-contour annotations to better separate lesion-specific signals from case-level background features.

Uncertainty-aware and probabilistic-attention methods may help quantify prediction reliability and reduce overconfidence in small or heterogeneous datasets [23]. Graph-based or geometric MIL approaches could also model spatial relationships between adjacent axial images and complement the bilateral information encoded by BAA-MIL [21]. These developments may be particularly relevant when extending the framework from a small number of preselected slices to complete volumetric examinations. Because malignant cases were already diagnosed breast cancer cases undergoing pretreatment staging CT, this dataset cannot assess the model’s ability to detect otherwise unsuspected cancers before their clinical diagnosis.

Future studies could additionally investigate medical-image-specific pretraining, self-supervised representation learning, and multimodal integration. Combining CT features with clinical, histopathological, or genomic information has been proposed as a means of supporting more comprehensive tumor characterization [16,27]. Advanced segmentation, denoising, and image-reconstruction methods may further improve the consistency of the model input [28]. However, these extensions would require substantially larger datasets and should be evaluated incrementally to determine whether they provide clinically meaningful improvements.

Ultimately, prospective evaluation should determine whether a refined BAA-MIL system can function as an opportunistic second-reader on routine chest CT. Such studies should compare model performance with radiologist interpretation, evaluate changes in sensitivity and recall recommendations, assess calibration and false-positive burden, and determine whether AI assistance improves clinically relevant outcomes without introducing unnecessary downstream investigations.

## 5. Conclusions

This proof-of-concept study developed and internally evaluated BAA-MIL, a bilateral-asymmetry-aware multiple-instance learning framework for case-level discrimination between breast cancer and non-malignant control examinations using preselected breast-containing axial chest CT images. Under the primary nested validation protocol, BAA-MIL achieved an ROC AUC of 0.547, with lower overall performance than the original exploratory non-nested analysis. This difference indicates that the original analysis may have overestimated generalization. The exploratory ablation analysis suggested that bilateral information, explicit asymmetry encoding, and multiple-instance aggregation may be useful components for future model development. However, the limited and uncertain performance under nested validation, small single-center case–control cohort, use of preselected images instead of complete CT volumes, and absence of external validation preclude immediate clinical application. Further evaluation using larger consecutive multicenter cohorts, complete DICOM examinations, improved lesion-level annotations, and prospective comparison with radiologists is required before this approach can be considered for opportunistic second-reader assessment of breast abnormalities on routine chest CT.

## Figures and Tables

**Table 1:** Layer-by-layer specification of the proposed BAA-MIL Model.

Component	Layer/Operation	Output Dimension	Details
Input	Paired unilateral crops per slice	2 × 3 × 224 × 224	One crop per breast side; grayscale PNGs converted to 3-channel RGB tensors
Shared encoder	Conv block 1	32 channels	3 × 3 Conv2D, stride 1, padding 1; BatchNorm2D; SiLU; MaxPool2D 2 × 2, stride 2;
Shared encoder	Conv block 2	64 channels	3 × 3 Conv2D, stride 1, padding 1; BatchNorm2D; SiLU; MaxPool2D 2 × 2, stride 2; Dropout2D 0.1
Shared encoder	Conv block 3	128 channels	3 × 3 Conv2D, stride 1, padding 1; BatchNorm2D; SiLU; MaxPool2D 2 × 2, stride 2; Dropout2D 0.2
Shared encoder	Conv block 4	256 channels	3 × 3 Conv2D, stride 1, padding 1; BatchNorm2D; SiLU; MaxPool2D 2 × 2, stride 2; Dropout2D 0.3
Feature pooling	Adaptive average pooling	256	AdaptiveAvgPool2D to 1 × 1, flattening
Bilateral descriptor	Mean feature	256	(fL + fR)/2
Bilateral descriptor	Asymmetry feature	256	
Descriptor input	Concatenation	512	[mean feature, absolute-difference feature]
Descriptor MLP	Linear	256	Linear 512-256
Descriptor MLP	Normalization/activation	256	LayerNorm, SiLU
Descriptor MLP	Dropout	256	Dropout rate 0.4
MIL pooling	Gated attention	256	Attention hidden size 128; tanh(Vh) × sigmoid(Uh) followed by linear projection to one attention logit per slice
Case classifier	Linear	2	Linear 256 to 2, softmax for non-malignant/malignant probability
Auxiliary slice head	Linear	2	Linear 256 to 2, used during training with weak case-level labels

**Table 2:** Architecture and training settings of the ablation variants.

Variant	Input	Feature Construction	Slice Aggregation	Training Settings
baseline_scratch	Individual unilateral crops	unilateral cropsCompact CNN encoder, no paired bilateral descriptor	Per-crop prediction; crop probabilities averaged to case level	Trained from scratch; Adam, learning rate 1 × 10^−3^, weight decay 1 × 10^−4^, cosine decay, 20 epochs, batch size 16 crops
single_mean	One unilateral crop per slice, **Breast_A**	Shared compact CNN encoder; no bilateral fusion; 256-dimensional crop feature	Mean pooling across valid slices	Same BAA-MIL training settings as final model
bilateral_mean	Breast_A and Breast_B crops per slice	Mean bilateral feature: (fA + fB)/2; no asymmetry descriptor	Mean pooling across valid slices	Same BAA-MIL training settings as final model
asymmetry_mean	Breast_A and Breast_B crops per slice	Shared compact CNN encoder for both crops; computes bilateral mean feature (fA + fB)/2 and absolute asymmetry feature	fA − fB	Concatenated into a 512-dimensional vector; descriptor MLPLinear 512-256, LayerNorm, SiLU, Dropout 0.4

**Table 3:** Case-level dataset composition and cohort characteristics.

Characteristic	Non-Malignant	Malignant	Total
Patients/CT examinations	40	49	89
CT indication	Heterogeneous non-breast-related clinical indications; not analyzed as separate subgroups	Pretreatment staging CT for known breast cancer	
Age, median years	65.5	62	64
Age range, years	47–81	30–84	30–84
Premenopausal	3/40	7/49	10/89
Postmenopausal	37/40	42/49	79/89
Breast density A	20/40	15/49	35/89
Breast density B	12/40	21/49	33/89
Breast density C	6/40	8/49	14/89
Breast density D	2/40	5/49	7/89
Histology: invasive carcinoma NST	N/A	44/49	—
Histology: invasive lobular carcinoma	N/A	2/49	—
Histology: mixed invasive lobular and ductal carcinoma	N/A	1/49	—
Histology: invasive mucinous carcinoma	N/A	1/49	—
Histology: ductal carcinoma in situ	N/A	1/49	—
Tumor laterality: left-sided	N/A	31/49	—
Tumor laterality: right-sided	N/A	18/49	—
Lesion size range, cm	N/A	0.7 × 0.8 × 0.8 to 9 × 12 × 11	—
Axial CT slices	148	156	304
Breast-region crops (both breasts)	296	312	608

**Table 4:** Case-level performance across the incremental architectural comparison, reported as mean ± standard deviation over the five held-out folds.

Model	Accuracy	Balanced Acc.	Sensitivity	Specificity	ROC AUC
baseline_scratch (per-crop CNN)	0.616 ± 0.103	0.617 ± 0.107	0.584 ± 0.224	0.650 ± 0.242	0.705 ± 0.164
single_mean (one breast, slice-MIL)	0.617 ± 0.086	0.621 ± 0.079	0.591 ± 0.168	0.650 ± 0.094	0.743 ± 0.122
bilateral_mean (both breasts, no asymmetry)	0.640 ± 0.113	0.643 ± 0.111	0.611 ± 0.201	0.675 ± 0.150	0.743 ± 0.120
+ asymmetry descriptor (mean)	0.694 ± 0.104	0.701 ± 0.111	0.602 ± 0.125	0.800 ± 0.187	0.741 ± 0.120
BAA-MIL (+ attention-MIL)	0.718 ± 0.121	0.726 ± 0.120	0.651 ± 0.178	0.800 ± 0.170	0.802 ± 0.099

**Table 5:** Paired patient-level bootstrap confidence intervals for ablation-model performance differences.

Comparison	Accuracy Delta	Balanced Accuracy Delta	Sensitivity Delta	Specificity Delta	ROC AUC Delta
bilateral_mean-single_mean	+5.6 pp [−3.4, +14.6]	+4.9 pp [−4.4, +13.7]	+12.2 pp [+1.9, +23.4]	−2.5 pp [−16.2, +10.5]	+11.2 pp [−5.7, +29.1]
asymmetry_mean-bilateral_mean	−5.6 pp [−14.6, +3.4]	−5.1 pp [−13.8, +4.0]	−10.2 pp [−22.5, +2.2]	0.0 pp [−11.9, +12.1]	−6.0 pp [−22.0, +8.7]
bamil_full-asymmetry_mean	+1.1 pp [−5.6, +7.9]	+1.2 pp [−5.4, +7.9]	0.0 pp [−8.3, +8.0]	+2.5 pp [−8.1, +14.3]	−2.1 pp [−10.1, +5.7]
bamil_full-baseline_scratch	−1.1 pp [−10.1, +6.7]	−2.2 pp [−11.1, +6.2]	+8.2 pp [0.0, +17.8]	−12.5 pp [−28.1, +2.4]	−10.5 pp [−23.0, +2.0]

**Table 6:** Primary nested patient-level cross-validation performance of the BAA-MIL model. Values are pooled out-of-fold case-level metrics. Bracketed values indicate 95% confidence intervals estimated by patient-level bootstrap resampling.

Model	Accuracy (95% CI)	Balanced Accuracy (95% CI)	Sensitivity (95% CI)	Specificity (95% CI)	ROC AUC (95% CI)
BAA-MIL, compact scratch CNN encoder	0.528 [0.427, 0.629]	0.555 [0.465, 0.637]	0.286 [0.160, 0.415]	0.825 [0.694, 0.930]	0.547 [0.420, 0.665]

**Table 7:** Backbone sensitivity analysis under nested patient-level cross-validation.

Backbone	Accuracy (95% CI)	Balanced Accuracy (95% CI)	Sensitivity (95% CI)	Specificity (95% CI)	ROC AUC (95% CI)
Compact scratch CNN encoder	0.528 [0.427, 0.629]	0.555 [0.465, 0.637]	0.286 [0.160, 0.415]	0.825 [0.694, 0.930]	0.547 [0.420, 0.665]
ImageNet-pretrained EfficientNet-B0 encoder	0.573 [0.472, 0.674]	0.573 [0.472, 0.675]	0.571 [0.435, 0.714]	0.575 [0.421, 0.725]	0.566 [0.451, 0.683]

**Table 8:** Exploratory inference-rule comparison (per-fold mean ± SD, 5-fold CV, same checkpoints).

Inference Rule	Accuracy	Balanced Acc.	ROC AUC
Case head,	0.684 ± 0.107	0.683 ± 0.112	0.764 ± 0.117
Slice-aggregation,	0.718 ± 0.121	0.726 ± 0.120	0.802 ± 0.099

**Table 9:** Summary of representative related studies in chest CT breast assessment and recent breast-cancer AI.

Study	Dataset Size	Imaging Modality	Relevance to Present Study
Park et al., 2023 [8]	84 patients with incidental breast cancer visible on prior chest CT	Contrast-enhanced chest CT	Establishes the clinical problem: incidental breast cancers on chest CT are often missed.
Koh et al., 2022 [10]	1170 preoperative chest CT scans: 1070 development, 100 internal test, 100 external test	Chest CT	Directly relevant CT-based deep-learning detection study; differs by using a larger dataset and detection framework.
Thomas et al., 2025 [11]	3541 chest CT examinations from a multi-institutional teleradiology practice	Chest CT plus radiology reports	Highly relevant to opportunistic detection of incidental breast lesions on chest CT, but focuses on workflow triage rather than case-level cancer classification.
Sahoo et al., 2024 [15]	123 HER2-positive breast-cancer patients from TCGA	H&E histopathological whole-slide images	Recent breast-cancer AI study, but not directly comparable because it uses histopathology and prognosis rather than chest CT classification.
Present study	89 patients: 49 malignant, 40 non-malignant; 304 selected axial images; 608 unilateral crops	Preselected breast-containing axial chest CT PNG images	Introduces bilateral-asymmetry-aware MIL for weakly supervised case-level CT classification.

## Data Availability

The raw data supporting the conclusions of this article will be made available by the authors on request.
